# Prognosis of men with penile metastasis and malignant priapism: a systematic review

**DOI:** 10.18632/oncotarget.23366

**Published:** 2017-12-18

**Authors:** Andrea Cocci, Oliver W. Hakenberg, Tommaso Cai, Gabriella Nesi, Lorenzo Livi, Beatrice Detti, Andrea Minervini, Girolamo Morelli, Marco Carini, Sergio Serni, Mauro Gacci

**Affiliations:** ^1^ Department of Urology, University of Florence, Florence, Italy; ^2^ Department of Urology, University Hospital Rostock, Rostock, Germany; ^3^ Department of Urology, Santa Chiara Hospital, Trento, Italy; ^4^ University of Florence, Florence, Italy; ^5^ Department of Urology, University of Pisa, Pisa, Italy

**Keywords:** penis metastasis, penile metastasis, penile cancer, penis cancer

## Abstract

**Introduction:** Metastases to the penis are rare, but can have severe consequences. The aim of this study was to systematically review the literature in order to gain more information on the presentation and prognosis of this metastatic disease.

We reviewed the literature relating to all case reports, series and reviews about penile metastasis, from 2003 to 2013, through a Medline search. We identified 63 articles and 69 patients.

Metastases were located on the root (38.8%), the shaft (38.8%) or the glans (22.2%) of the penis. The diagnosis of penile metastasis was made after the primary cancer had been diagnosed. The most common presentation was a single small penile nodule. Ten patients reported priapism. The median survival time after diagnosis of penile metastasis was 10 months (range 6-18 months). A Kaplan-Meier analysis has shown that the patients presenting with priapism and those with metastases from non-urologic tumors have a significantly worse prognosis (age adjusted Log Rank: p=0.037 for priapism vs. no priapism and p=0.045 for urologic vs. non urologic).

There are prognostic differences based on the presentation of penile metastases. Survival is substantial and treatment should therefore take into account symptoms improvement and quality of life.

## INTRODUCTION

Although penile metastasis is relatively rare, its management presents a challenging problem. The first description of a penile metastasis was published in 1870 [1] and the first extensive review of the problem in 1961 by Abeshouse et al. [2]. Since then, about 460 additional cases have been reported in literature.

The most common primary malignancies with penile metastases are urogenital cancers (69%) followed by cancers from gastrointestinal origin (19%) [1, 2, 3, 4, 5, 6, 7, 8, 9, 10, 11, 12]. The clinical manifestations are heterogeneous, ranging from penile nodules to masses, with or without ulceration, obstructive or irritative urinary symptoms, hematuria or priapism. It has been suggested that the occurrence of priapism as a consequence of malignancy may be a prognostic factor to take into account [2, 3, 5, 10, 12].

Priapism can be defined as a prolonged penile erection in absence of sexual stimulation, usually caused by hematological diseases such as sickle cell anemia, leukemia or polycythemia, pelvic thrombosis or thrombophlebitis, by neurological diseases or by extensive pelvic tumors with or without penile metastasis [2, 4, 5, 8]. Various mechanisms for priapism secondary to cancer have been suggested, including tumor infiltration of the corpora cavernosa or blockage of the cavernous venous drainage system [1]. Metastases to the penis mimicking priapism are extremely rare, especially in the absence of a disseminated disease.

Most cancers leading to penile metastasis are from pelvic organs: prostate and bladder followed by colon of the recto-sigmoid region. Priapism resulting from such metastases will often be clinically overlooked and therefore difficult to treat. Whether or not the occurrence of priapism secondary to penile metastases can be one of the major prognostic factors remains unknown and the aim of this systematic review is to clarify this question.

## MATERIALS AND METHODS

### Evidence acquisition

We conducted a search of the English language literature, ranging from 2003 to January 2013, using the Medline database of the US National Library of Medicine (http://www.ncbi.nlm.nih.gov/pubmed) and the Google Scholar database. The Medline search was carried out by using the following Medical Subject Headings (MESH) and free text terms: penile, and metastasis were combined with the terms ‘treatment, clinical manifestations, therapy’ and then limited to ‘humans, male and young adult, 19-24 years’.

**Figure 1:**
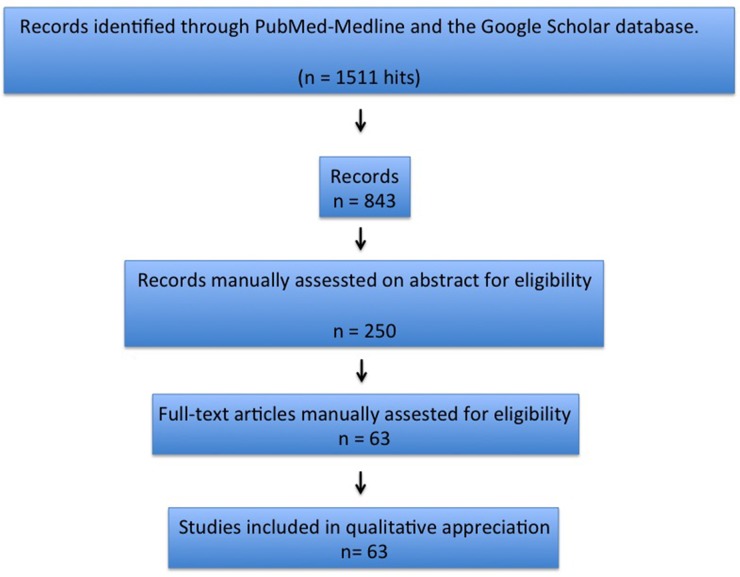
Articles’ selection process

Abstracts were excluded if subsequently followed by extended articles. Overlapping reports were not considered because of redundant information. From the initial literature search yielding 843 unique citations, a total of 63 papers were selected to review. Out of these 63 papers, 69 patients and their data were used for the analysis [1–51]. The Prisma Statement was used to perform an accurate research check-list and report (Figure 1).

### Statistical analysis

For the null hypothesis, we assumed that there was no difference in terms of outcome between all clinical, pathological and instrumental parameters in patients affected by secondary penile malignancy. Fisher's exact test and chi-square test were used to assess the significance of differences between parameters, with p<0.05 considered the cut-off for significance. Categorical variables were presented as percentages and were compared using χ^2^ analysis. Continuous variables were presented as the mean ± standard deviation and were compared using Student's *t*-test or the Mann–Whitney *U*-test.

Relative Risks and 95% confidence intervals were estimated by applying log-binomial regression and Cox regression analysis with a constant in the time variable. Moreover, difference in survival were assessed by Kaplan Meyer survival curves (and age adjusted log rank). All reported p values are 2-sided. Statistical analyses were performed using SPSS 11.0 for Apple-Macintosh (SPSS, Chicago, Illinois).

## RESULTS

The population for analysis consisted of patients with an age range of 57 to 92 years and a mean follow-up of 15.6 months (range 5-30). The clinical characteristics of the patients are given in Table 1.

**Table 1:** Clinical characteristics of the patients

Primary tumour		Prostate	TCC	Other
No. of cases		17	13	39
Age	mean (years)	72.8 (40-92)	63 (59-73)	65.3 (57-73)
Localization	root (n.)	3	3	2
	Shaft	2	3	3
	Glans	3	0	1
	unknown	9	7	33
Diameter	mean (cm)	2.2 (1-4)	2.6 (1-5)	1 (0.5-2)
Presentation	painless nodule	10	5	21
	Priapism	2	3	5
	other symptoms	2	3	5
	Unknown	3	2	7
Timing of diagnosis vs. diagnosis of primary tumor	Synchronous	1	1	2
	Metachronous	8	1	4
	Unknown	8	11	33
Treatment	LHRH analogue	4		
	total penectomy	1	1	
	radical cystoprostatectomy		1	
	conservative penile sparing therapy		1	
	intravenous temsirolimus			1
	None	1		
	Unknown treatment	11	10	38
Cancer specific survival	mean % died	66.7	83.3	80
	mean follow-up time (months)	16.4	19.2	11.2

### Clinical presentation and treatment

Penile metastases were located at the root (38.8%), the shaft (38.8%) or the glans (22.2%) of the penis. Five patients had multiple penile metastatic lesions. In four patients the diagnosis of penile metastasis was synchronous with the diagnosis of the primary tumor, but metachronous in the majority. The most common form of presentation was a single small painless nodule of 1-2 cm in diameter. Ten patients presented with priapism secondary to penile metastasis.

### Pathological considerations

In 33 cases (47.8%), the primary cancer originated from the urogenital tract, prostate cancer in 17 cases (24,6%), bladder cancer in 13 (18,8%), renal cancer in 2 (2,9%) and seminal vesicle cancer in one patient (1,5%). 36 cases (52,2%) originated from other cancers such as skin, lung, colorectal, esophagus, tongue, jaw, thyroid, testis, lymphoma and glomangiosarcoma.

### Survival analysis

**Figure 2:**
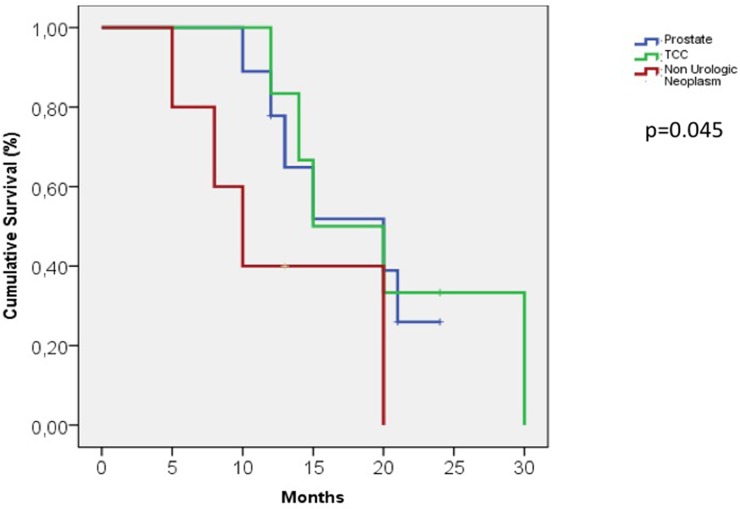
Kaplan-Meier curve, patients with metastases from urological and non-urological tumors

**Figure 3:**
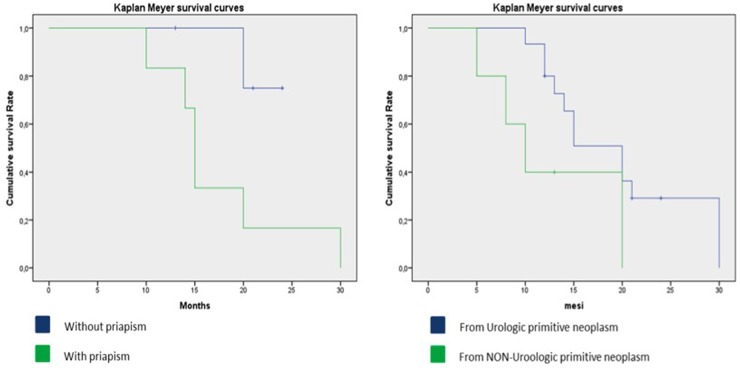
Kaplan-Meier curve, details of patients with metastases from urological and non-urological tumors and presence of malignant priapism

The median cancer-specific survival time for men with penile metastasis was 14.5 months (range 5-30). The Kaplan-Meier curve analysis showed that patients with metastases from non-urological tumors generally seemed to have a poorer prognosis than those with tumours of urological origin (Figure 2) and that patients presenting with malignant priapism had a worse prognosis than those without priapism (Figure 2). Thus, patients with priapism as the presenting symptom from a metastasis originating from a non-urological malignancy had a worse prognosis compared to those with metastases from urological malignancies and without priapism (age adjusted log rank p=0.045 for urological vs. non-urological and p=0.037 for priapism vs. no priapism) (Figure 3).

30 patients with urological metastases (43%) had a median cancer specific survival time of 18 months compared to 30 patients with non urological metastases (57%) who had a median cancer specific survival time of 11 months.

**Figure 4:**
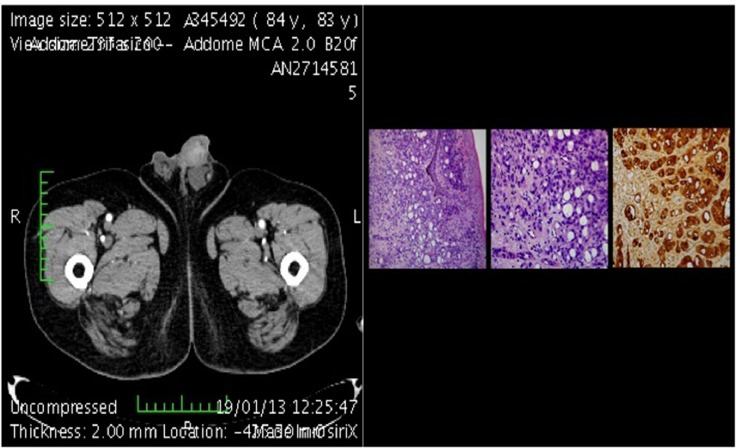
CT-scan of metastasis plus histological section

10 patients presented with priapism as the first symptom (5 from urological and 5 from non-urological cancers). Patients with priapism from urological cancer had a median cancer specific survival time of 30 months, patients with priapism from non-urological cancer had a median cancer specific survival time of 15 months (Figure 4).

## DISCUSSION

Penile metastases are relatively rare and usually occur in the context of more widespread disseminated disease. Therefore, the prognosis is significantly poor. However, little more than this is known about the problem.

As penile metastases are only reported in case reports or small case series there will never be reliable evidence from larger trials from prospective series. Thus, the only evidence that can be used to gather information about penile metastases is from case reports. Therefore, we analysed the available literature on case reports of penile metastases in order to gain more information.

Our analysis is the most comprehensive systematic review of the topic with a clinically relevant number of cases. Moreover, we performed our analysis on all cases of penile metastasis without excluding patients based on the site of the primary malignancy or the presentation. This approach has, of course, several limitations as its nature is retrospective, making it therefore impossible to assess data other than those reported by the primary authors. However, we excluded patients with incomplete clinical or pathological data, i.e. when survival time or pathology of the primary tumor were not given. Another limitation is that it was impossible to draw any conclusion on the impact of the treatment from the case reports we analysed.

Thus, our limited analysis shows that penile metastases presenting with priapism have a very poor prognosis, especially if the primary tumor is not of prostatic or bladder origin. However, based on the published cases, the mean survival time is 14 months and this data has to be considered in view of a possible disseminated disease, which could in turn cause other symptoms.

Our finding that malignant priapism is associated with a poorer prognosis in patients with penile metastases is consistent with other previous reports. Whilst penile metastases most commonly appear as an infiltrative lesion or nodule, up to 40% of cases reported intermittent or continuous malignant priapism. This was first described in 1938 by Peacock.

A literature review by Lin YH et al based on reports from 2006 to 2011 suggested that the true incidence of penile metastasis may be higher given that 12% of penile metastasis may be asymptomatic and discovered only at autopsy. They also suggested that most cases of malignant priapism would be low-flow priapism due to neoplastic invasion of cavernous sinuses and venous system [2]. While this theory seems to be highly plausible, some authors have suggested that malignant priapism may also be due to high flow in some cases. Dubocq et al used doppler ultrasound and have found evidence for this theory in their cases. Differences in the pathophysiology of malignant priapism may also be related to the mode of the metastatic spread. Whilst urological cancers may also invade the penile cavernous bodies directly, non-urological metastases will occur from lymphatic or hematogenous spread.

We calculated a mean survival time of the reported cases of 14 months. Lin YH et al. reported an average cancer-specific survival time of 9 months with an overall survival time of under 18 months. According to our analysis, survival time is over one year and therefore is a substantial data for planning a treatment. Whilst the treatment approach is commonly palliative, this may be questioned in view of the survival time. According to our review, in patients with better prognostic indicators (urological cancer, no priapism), the efforts of the treatment should be on prolonging the survival time and enhancing the quality of life.

## CONCLUSIONS

Whilst penile metastasis is rare and is only one of the manifestations of a disseminating cancer, there are differences in prognosis between patients presenting with or without priapism and those with urological or non-urological primary cancers. Cancer-specific survival time is on average substantial with over one year and treatment should take this data into consideration.
